# Correlation Between Antimicrobial Resistance, Virulence Determinants and Biofilm Formation Ability Among Extraintestinal Pathogenic *Escherichia coli* Strains Isolated in Catalonia, Spain

**DOI:** 10.3389/fmicb.2021.803862

**Published:** 2022-01-11

**Authors:** Victoria Ballén, Yaiza Gabasa, Carlos Ratia, Melany Sánchez, Sara Soto

**Affiliations:** ISGlobal, Hospital Clínic—Universitat de Barcelona, Barcelona, Spain

**Keywords:** *Escherichia coli*, antimicrobial resistance, virulence, biofilm, colibactin

## Abstract

*Escherichia coli* is a well-characterized bacterium highly prevalent in the human intestinal tract and the cause of many important infections. The aim of this study was to characterize 376 extraintestinal pathogenic *E. coli* strains collected from four hospitals in Catalonia (Spain) between 2016 and 2017 in terms of antimicrobial resistance, siderophore production, phylogroup classification, and the presence of selected virulence and antimicrobial resistance genes. In addition, the association between these characteristics and the ability to form biofilms was also analyzed. The strains studied were classified into four groups according to their biofilm formation ability: non-biofilm formers (15.7%), weak (23.1%), moderate (35.6%), and strong biofilm formers (25.6%). The strains were highly resistant to ciprofloxacin (48.7%), trimethoprim-sulfamethoxazole (47.9%), and ampicillin (38%), showing a correlation between higher resistance to ciprofloxacin and lower biofilm production. Seventy-three strains (19.4%) were ESBL-producers. However, no relationship between the presence of ESBL and biofilm formation was found. The virulence factor genes *fimH* (92%), *pgaA* (84.6%), and *irp1* (77.1%) were the most prevalent in all the studied strains. A statistically significant correlation was found between biofilm formation and the presence of *iroN*, *papA*, *fimH*, *sfa*, *cnf*, *hlyA*, *iutA*, and colibactin-encoding genes *clbA*, *clbB, clbN*, and *clbQ*. Interestingly, a high prevalence of colibactin-encoding genes (19.9%) was observed. Colibactin is a virulence factor, which interferes with the eukaryotic cell cycle and has been associated with colorectal cancer in humans. Most colibactin-encoding *E. coli* isolates belonged to phylogroup B2, exhibited low antimicrobial resistance but moderate or high biofilm-forming ability, and were significantly associated with most of the virulence factor genes tested. Additionally, the analysis of their clonal relatedness by PFGE showed 48 different clusters, indicating a high clonal diversity among the colibactin-positive strains. Several studies have correlated the pathogenicity of *E. coli* and the presence of virulence factor genes; however, colibactin and its relationship to biofilm formation have been scarcely investigated. The increasing prevalence of colibactin in *E. coli* and other Enterobacteriaceae and the recently described correlation with biofilm formation, makes colibactin a promising therapeutic target to prevent biofilm formation and its associated adverse effects.

## Introduction

*Escherichia coli* is a well-characterized bacterium which plays an essential role in the human microbiome. Nevertheless, some strains are responsible for intestinal and extraintestinal infections. Extraintestinal pathogenic *E. coli* (ExPEC) strains are commonly implicated in a variety of infectious diseases occurring in either the community or healthcare settings worldwide, resulting in high economic and social costs (Beloin et al., 2008; Blount, 2015; Manges et al., 2019).

Additionally, *E. coli* can form aggregates and attach to solid surfaces, forming complex structures called biofilms. Bacteria in biofilms secrete various components such as extracellular polymeric substances (EPS), which protect the bacterial community from host immunity and the effects of antibiotics, complicating the infection (Sharma et al., 2016).

In addition to the ability to form biofilms, the ExPEC group has many virulence factor genes (VFGs) encoding adhesins, toxins, siderophores, capsules, and invasins, which are often located into pathogenicity islands (PAIs), plasmids, and other mobile genetic elements (Sarowska et al., 2019). Some of these VFGs can promote biofilm formation. Among the well-characterized VFGs correlated with biofilm formation, type I fimbriae, curli fimbriae, and flagella are the most studied. The *fimA* gene encodes the major subunit of type I fimbriae, which are known to be involved in the first step of biofilm formation (Beloin et al., 2004). Curli fimbriae are encoded by curli-specific genes (*csg*) and are involved in adhesion to surfaces and invasion of eukaryotic host cells (Van Gerven et al., 2018). Flagella play an important role not only in cell motility but also in surface adhesion (Friedlander et al., 2015). Some studies suggest that deletion of some of these genes leads to a reduction in the ability to form biofilm (Guttenplan and Kearns, 2013; Smith et al., 2017).

One of the most recently studied VFGs is colibactin, a secondary metabolite encoded in the genomic island *pks*, which interferes with the eukaryotic cell cycle and has been linked to colorectal cancer in humans (Dziubańska-Kusibab et al., 2020). Interestingly, this PAI is commonly observed among *E. coli* strains belonging to phylogroup B2, including ExPEC, and has been found in isolates from the intestinal microbiota, septicemia, neonatal meningitis, and urinary tract infections (UTIs) (Faïs et al., 2018; Dziubańska-Kusibab et al., 2020).

The aim of this study was to characterize 376 ExPEC strains in terms of antimicrobial resistance, biofilm formation, siderophore production, phylogroup classification, and presence of selected virulence and antimicrobial resistance genes. The association between these features and the ability to form biofilm was also analyzed. Understanding the VFGs that correlate with biofilm production is needed, as these could be considered targets for developing new antimicrobial therapies.

## Materials and Methods

### Bacterial Strains

A total of 376 ExPEC strains were collected from four hospitals in Catalonia, Spain, between 2016 and 2017. Of these, 132 were isolated from blood, 60 from respiratory samples (13 sputum, 12 bronchoalveolar aspirates, 4 tracheal samples, 2 pleural fluid, and 29 non-classified respiratory samples) and 184 from urine (1 from a urinary catheter and 183 from midstream urine). Strains were identified by matrix-assisted laser desorption ionization–time-of-flight mass spectrometry (MALDI-TOF) (Bruker Daltonik GmbH, Bremen, Germany). The modified score values suggested by the manufacturer were used: a score ≥ 2.3 meant species identification; a score between 2.0 and 2.299 meant genus identification and probable species identification; a score between 1.7 and 1.9 meant probable genus identification; and a score < 1.69 meant non-reliable identification. Only strains with a score ≥ 2.3 classified as *E. coli* were included in the study. After that, the strains were stored in Skim Milk (Becton Dickinson) at −80°C.

### Biofilm Formation and Quantification

To determine biofilm formation, we performed a protocol previously developed by our group (Cepas et al., 2020). Briefly, strains were cultured in Luria Bertani (LB) agar (Miller’s LB AGAR, Condalab) for 18 – 24 h at 37°C. Then, the cell suspension was prepared in 10 mL LB broth and incubated for 18 – 24 h at 37°C with shaking (180 rpm). After incubation, each culture was diluted 1:100 in M63 medium [13.5 g/L KH_2_PO_4_, 2 g/L (NH_4_)_2_SO_4_, 5.0 × 10^–4^ g/L FeSO_4_, 1 mL 1 M MgSO_4_.7H_2_O], supplemented with 0.25% glucose and tested in 96-well flat-bottomed non-treated polystyrene microtiter plates with lids (Nunc^®^ Edge 2.0, VWR International, Barcelona, Spain) at 30°C for 48 h under static conditions.

The supernatant was then removed and the biofilms were washed once with 1x PBS and dried at 65°C. The plates were stained with crystal violet 2% (CV) for 10 min, washed with 1x PBS, and dried at 65°C. The CV was resuspended with glacial acetic acid 33%, and the biomass was quantified by measuring the optical density at 580 nm using a microplate reader (EPOCH 2 microplate reader; BioTek, VT). The experiment was performed in three technical and biological replicates, and the results were interpreted according to the criteria of Stepanović et al. (2007). Thus, the strains were classified as non-biofilm formers (OD ≤ 0.150), weak biofilm formers (≥ 0.151 OD ≤ 0.300), moderate biofilm formers (≥ 0.301 OD ≤ 0.60), or strong biofilm formers (OD ≥ 0.601). *Escherichia coli* ATCC 25922 was used as positive control, and M63 broth without bacterial inoculum was used as negative control.

### Antimicrobial Susceptibility Testing

Antimicrobial resistance profiling was performed using the most representative antimicrobial agents from the different antibiotic families, which are of great clinical and epidemiological relevance. Kirby-Bauer disk diffusion or broth microdilution methods (in the case of colistin) were done according to Clinical and Laboratory Standards Institute (CLSI) guidelines (CLSI, 2020). *Escherichia coli* ATCC 25922 was used as a control strain. The antimicrobials tested by disk diffusion were: amoxicillin/clavulanate (20/10 μg), ampicillin (10 μg), aztreonam (30 μg), cefepime (30 μg), cefotaxime (30 μg), ceftazidime (30 μg), chloramphenicol (30 μg), ciprofloxacin (5 μg), fosfomycin (200 μg/50 μg of glucose-6-phosphate), gentamicin (10 μg), imipenem (10 μg), meropenem (10 μg), and trimethoprim-sulfamethoxazole (1.25/23.75 μg) (Becton Dickinson). Extended-spectrum beta-lactamases were screened by the ESBL test following the CLSI guidelines (CLSI, 2020). Isolates were classified as susceptible, resistant to 1 or 2 antimicrobial categories, multidrug-resistant (MDR) if resistant to at least one agent in ≥ 3 antimicrobial categories; or extensively drug-resistant (XDR), if resistant to at least one agent in all but two or fewer antimicrobial categories (Magiorakos et al., 2012).

### Identification of Antimicrobial Resistance Genes

Polymerase chain reaction (PCR) assays were performed using the supernatant of a boiled cell suspension of each isolate as DNA template. β-lactamase-encoding genes *bla*_*SHV–*1_, *bla*_*TEM–*1_ and the five major groups *bla*_*CTX–M:*_ *bla*_*CTX–M–*1_, *bla*_*CTX–M–*2_, *bla*_*CTX–M–*8_, *bla*_*CTX–M–*9_, and *bla*_*CTX–M–*25_ were detected, as well as genes conferring resistance to sulfonamides (*sul1* and *sul2*), quinolones [*qnrB* and *aac(6’)-lb-cr*] and colistin (*mcr-1*, *mcr-2*, *mcr-3*, *mcr-4* and *mcr-5*). Previously characterized strains from our laboratory collection were used as positive controls of the different genes in the corresponding PCR experiments. Water was used as negative control. The PCR products from the strains were sequenced (Genewiz). The obtained sequences were compared with those of the corresponding genes available in the GenBank. Primer sequences (Condalab, Spain) used in the study of antimicrobial resistance genes are listed in Table 1.

**TABLE 1:** Primers to detect antimicrobial resistance genes.

Target gene	Primer sequence (5′ → 3′)	Melting Temperature (Tm °C)	Product size (bp)	References
*bla*_*TEM–*1_ – F	TCGCCGCATACACTATTCTCAGAATGA	53	445	Monstein et al., 2007
*bla*_*TEM–*1_ – R	ACGCTCACCGGCTCCAGATTTAT			
*bla*_*SHV–*1_ – F	ATGCGTTATATTCGCCTGTG	49	747	Wiegand et al., 2007
*bla*_*SHV–*1_ – R	TGCTTTGTTATTCGGGCCAA			
*bla*_*CTX–M–*1_ – F	AAAAATCACTGCGCCAGTTC	52	415	Woodford et al., 2006
*bla*_*CTX–M–*1_ – R	AGCTTATTCATCGCCACGTT			
*bla*_*CTX–M–*2_ – F	CGACGCTACCCCTGCTATT	52	552	Woodford et al., 2006
*bla*_*CTX–M–*2_- R	CCAGCGTCAGATTTTTCAGG			
*bla*_*CTX–M–*9_ – F	CAAAGAGAGTGCAACGGATG	52	205	Woodford et al., 2006
*bla*_*CTX–M–*9_ – R	ATTGGAAAGCGTTCATCACC			
*bla*_*CTX–M–*8_ – F	TCGCGTTAAGCGGATGATGC	52	666	Woodford et al., 2006
*bla*_*CTX–M–*8_ – R	AACCCACGATGTGGGTAG			
*bla*_*CTX–M–*25_ – F	GCACGATGACATTCGGG	52	327	Woodford et al., 2006
*bla*_*CTX–M–*25_- R	AACCCACGATGTGGGTAG			
*bla*_*CTX–M–*15/28_ – F	ATAAAACCGGCAGCGGTG	55	483	Leflon-Guibout et al., 2004
*bla*_*CTX–M–*15/28_- R	GAATTTTGACGATCGGGG			
*bla*_*CTX–M–*14/27_ – F	CGCTTTATGCGCAGACGA	57	785	Pai et al., 2001
*bla*_*CTX–M–*14/27_- R	GATTCTCGCCGCTGAAGC			
*sul1* – F	CTTCGATGAGAGCCGGCGGC	63	436	Guerra et al., 2004
*sul1* – R	GCAAGGCGGAAACCCGCGCC			
*sul2* – F	TCAACATAACCTCGGACAGT	55	707	Guerra et al., 2004
*sul2* – R	GATGAAGTCAGCTCCACCT			
*aac(6’)-Ib* – *cr* –F	TTGCGATGCTCTATGAGTGGCTA	60	482	Park et al., 2006
*aac(6’)-Ib* – *cr* – R	CTCGAATGCCTGGCGTGTTT			
*qnrB* – F	GATCGTGAAAGCCAGAAAGG	52	469	Robicsek et al., 2006
*qnrB* – R	ACGATGCCTGGTAGTTGTCC			
*mcr-1 –* F	ATGCCAGTTTCTTTCGCGTG	59	502	Lescat et al., 2018
*mcr-1 –* R	TCGGCAAATTGCGCTTTTGGC			
*mcr-2 –* F	GATGGCGGTCTATCCTGTAT	59	379	Lescat et al., 2018
*mcr-2 –* R	AAGGCTGACACCCCATGTCAT			
*mcr-3 –* F	ACCAGTAAATCTGGTGGCGT	59	296	Lescat et al., 2018
*mcr-3 –* R	AGGACAACCTCGTCATAGCA			
*mcr-4 –* F	TTGCAGACGCCCATGGAATA	59	207	Lescat et al., 2018
*mcr-4 –* R	GCCGCATGAGCTAGTATCGT			
*mcr-5 –* F	GGACGCGACTCCCTAACTTC	59	608	Lescat et al., 2018
*mcr-5 –* R	ACAACCAGTACGAGAGCACG			

### Virulence Determinants Detection

Virulence factor genes encoding for adhesins (*fimH-1*, *sfa*, *papA*, *pgaA*), siderophores (*iroN*, *iutA*, *irp-1*, *iucA*) and toxins (*hlyA*, *cnf-1*, *clbA clbB*, *clbN* and *clbQ*) were detected by PCR. To determine the presence of the complete *pks* genomic island, primers for the four most representative genes were used: *clbA* and *clbQ* as flanking primers, and *clbB* and *clbN* as internal primers (Johnson et al., 2008; Dubois et al., 2010; Suresh et al., 2018). The primer sequences (Condalab, Spain) used for the detection of the different VFGs are listed in Table 2. Previously characterized strains carrying the different VFGs were used as positive controls. Water was used as negative control.

**TABLE 2:** Primers to detect virulence factor genes.

Target gene	Primer sequence (5′ → 3′)	Melting temperature (Tm °C)	Product size (bp)	Function	References
*iroN* – F	AAGTCAAAGCAGGGGTTGCCCG	56	827	Siderophore uptake transmembrane transporter activity	Johnson et al., 2000
*iroN* – R	GACGCCGACATTAAGACGCAG				
*irp1* – F	GGCGTCTCCTCCTTTGGTATT	60	1729	Gene encoding for an iron regulatory protein	Xu et al., 2000
*irp1* – R	GTGATTCCCGCTGTTGATGTT				
*iucA* – F	AGTCTGCATCTTAACCTTCA	56	1100	Gene encoding for an aerobactin	Guerrieri et al., 2019
*iucA* – R	CTCGTTATGATCGTTCAGAT				
*papA* – F	ATGGCAGTGGTGTCTTTTGGTG	62	717	Fimbrial major pilin protein precursor	Johnson and Stell, 2000
*papA* – R	CGTCCCACCATACGTGCTCTTC				
*fimH* – F	CAGCGATGATTTCCAGTTTGTGTG	59	461	Type 1 fimbrin D-mannose specific adhesin precursor	Sáez-López et al., 2017
*fimH* – R	TGCGTACCAGCATTAGCAATGTCC				
*sfa* – F	CTCCGGAGAACTGGGTGCATCTTAC	65	410	S fimbriae	Houdouin et al., 2006
*sfa* – R	CGGAGGAGTAATTACAAACCTGGCA				
*cnf-1* – F	AAGATGGAGTTTCCTATGCAGGAG	56	498	Cytotoxic necrotizing factor	Takahashi et al., 2006
*cnf-1* – R	CATTCAGAGTCCTGCCCTCATTATT				
*hlyA* – F	AACAAGGATAAGCACTGTTCTGGC	59	1177	Hemolysin	Johnson and Stell, 2000
*hlyA* – R	ACCATATAAGCGGTCATTCCCGTCA				
*iutA* – F	GGCTGGACATCATGGGAACTGG	60	300	Ferric aerobactin receptor precursor	Johnson and Stell, 2000
*iutA* – R	CGTCGGGAACGGGTAGAATCG				
*pgaA* – F	GGCTTTGAAACTTCTTACTGC	60	209	Poly-beta-1,6-N-acetyl-D-glucosamine export protein	Shrestha et al., 2019
*pgaA* – R	CCTGTTTATCTTGCCCGGCC				
*clbQ* – F	CTTGTATAGTTACACAACTATTTC	54	821	Colibactin biosynthesis thioesterase ClbQ	Morgan et al., 2019
*clbQ* – R	TTATCCTGTTAGCTTTCGTTC				
*clbA* – F	CTAGATTATCCGTGGCGATTC	54	1002	Colibactin biosynthesis phosphopantetheinyl transferase ClbA	Morgan et al., 2019
*clbA* – R	CAGATACACAGATACCATTCA				
*clbB* – F	GATTTGGATACTGGCGATAACCG	54	579	Colibactin hybrid non-ribosomal peptide synthetase/type I polyketide synthase ClbB	Johnson et al., 2008
*clbB* – R	CCATTTCCCGTTTGAGCACAC				
*clbN* – F	GTTTTGCTCGCCAGATAGTCATTC	54	733	Colibactin non-ribosomal peptide synthetase ClbN	Johnson et al., 2008
*clbN* – R	CAGTTCGGGTATGTGTGGAAGG				

### Siderophore Assay

Siderophores production was determined according to the protocol described by Schwyn and Neiland (Schwyn and Neilands, 1987). Briefly, bacterial strains were cultured on chrome azurol S (CAS) (VWR) and hexadecyltrimethylammonium bromide (HDTMA) plates (Fisher Scientific). If a bacterium excretes a siderophore that removes iron from the dye complex, the color of the agar changes from blue to orange. *Acinetobacter baumannii* ATCC19606 was used as positive control.

### Phylogroup Assignment Method

*Escherichia coli* strains were classified into seven phylogroups (A, B1, B2, C, D, E, and F) according to the PCR method designed by Clermont et al. (2013). Strains belonging to our group and whose phylogroup was previously identified were used as controls.

### Typing of Colibactin-Positive Strains by Pulsed-Field Gel Electrophoresis

Due to the high prevalence of colibactin among the studied strains, we decided to analyze their clonal relationship by pulsed-field gel electrophoresis (PFGE) of *Xba*I-digested DNA, following the protocol described by Durmaz et al. (2009). The profiles obtained were compared using the InfoQuestTM FPv.5.4 software (Bio-Rad Laboratories) and the unweighted pair group method with arithmetic mean to create dendrograms based on Dice’s similarity coefficient. Isolates were clustered together if their similarity index was ≥ 85%.

### Statistical Analysis

Statistical analyses were performed using IBM SPSS Statistics for Windows software, version 21.0. The Chi-square test was used to evaluate correlations among variables. p-values < 0.05 were considered statistically significant.

## Results

### Biofilm Formation and Quantification

*Escherichia coli* strains were analyzed for biofilm formation using M63 broth. Fifty-nine strains (15.7%) were classified as non-biofilm-forming isolates, 87 (23.1%) were classified as weakly biofilm-forming, 134 (35.6%) as moderately biofilm-forming, and 96 (25.6%) as strongly biofilm-forming strains.

**FIGURE 1:**
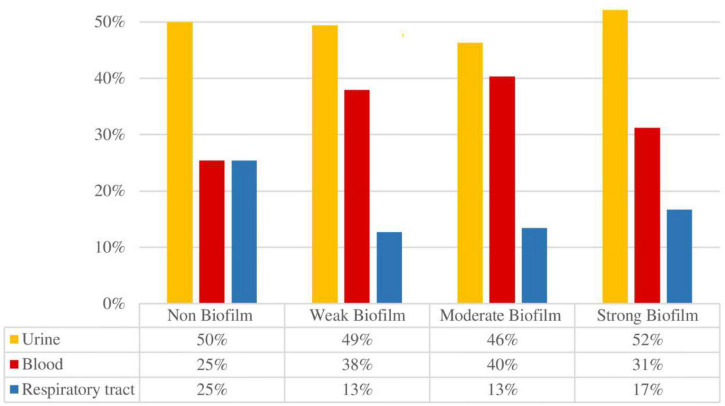
Biofilm formation according to the origin of the isolates.

Furthermore, the ability to form biofilm was investigated according to the origin of the isolates (urine, blood or respiratory tract) as shown in Figure 1; however, no correlation between strain source and biofilm formation ability was found (*p* > 0.05).

### Antimicrobial Susceptibility Testing

**FIGURE 2:**
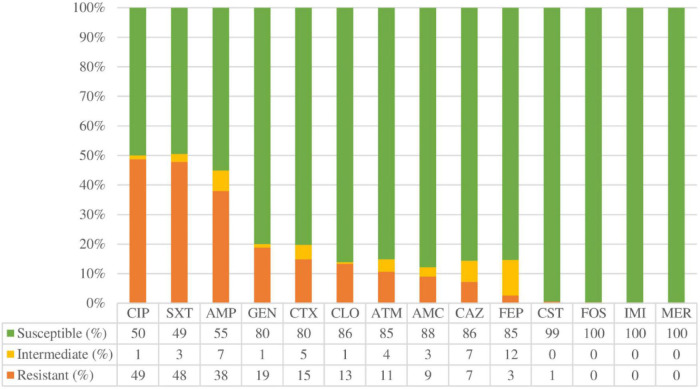
Overall antimicrobial resistance rates expressed in percentage (%). AMC: Amoxicillin/clavulanic acid, AMP: Ampicillin, ATM: Aztreonam, CLO: Chloramphenicol, CAZ: Ceftazidime, CIP: Ciprofloxacin, CST: Colistin, CTX: Cefotaxime, FEP: Cefepime, FOS: Fosfomycin, GEN: Gentamicin, IMI: Imipenem, MER: Meropenem, SXT: Trimethoprim-sulfamethoxazole.

**FIGURE 3:**
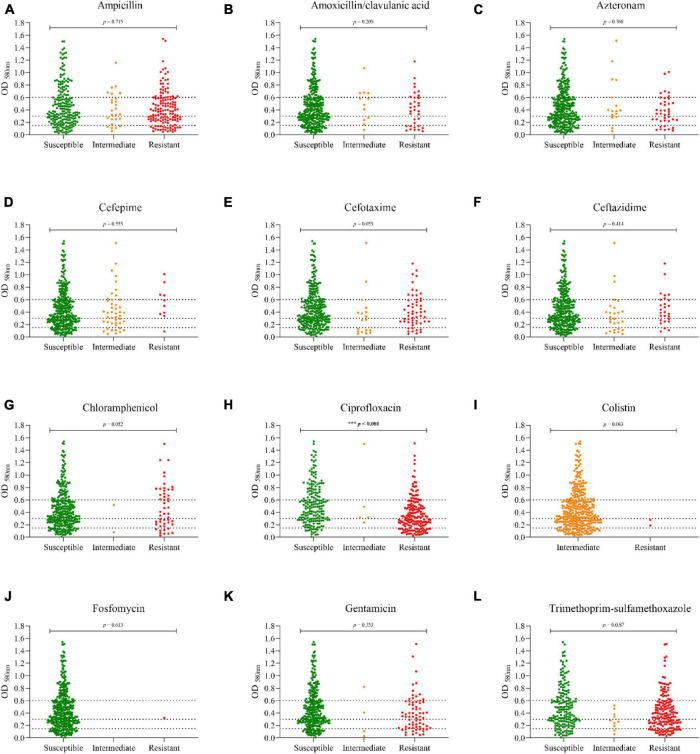
Distribution of biofilm formation among the different antibiotic resistance profiles. **(A)** Ampicillin; **(B)** Amoxicillin/Clavulanic acid; **(C)** Aztreonam; **(D)** Cefepime; **(E)** Cefotaxime; **(F)** Ceftazidime; **(G)** Chloramphenicol; **(H)** Ciprofloxacin; **(I)** Colistin; **(J)** Fosfomycin; **(K)** Gentamicin; **(L)** Trimethoprim-sulfamethoxazole. The dotted lines indicate the cut-off points for each biofilm category [non-biofilm formers (OD ≤ 0.150), weak biofilm formers (≥ 0.151 OD ≤ 0.300), moderate biofilm formers (≥ 0.301 OD ≤ 0.60), or strong biofilm formers (OD ≥ 0.601)].

The percentages of antimicrobial resistance are shown in Figure 2. Overall, the highest percentages of antibiotic resistance were observed with ciprofloxacin (48.7%), trimethoprim-sulfamethoxazole (47.9%), and ampicillin (38%). Only two strains were resistant to colistin (0.5%), and one to fosfomycin (0.3%). None was resistant to carbapenems (imipenem or meropenem). To determine whether biofilm formation correlates with antimicrobial resistance, the different categories of biofilm formation were compared with resistance profiles as shown in Figure 3. Non-biofilm-forming strains showed higher percentages of resistance to ciprofloxacin (74.6%) compared to biofilm-forming strains (Figure 3H). Thus, the higher the resistance to ciprofloxacin, the lower the biofilm production ability (*p* < 0.0001).

**FIGURE 4:**
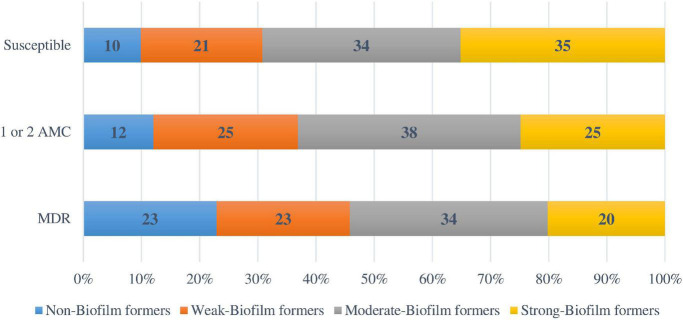
Biofilm formation according to the antimicrobial resistance classification. AMC: Antimicrobial categories; MDR: Multidrug-resistant.

Regarding the antimicrobial resistance classification, 91 strains (24.2%) were susceptible to all the antimicrobial categories tested, 142 (37.8%) were resistant to one or two antimicrobial categories, 143 (38%) isolates were MDR, and none was XDR. The percentages for biofilm formation by antimicrobial resistance classification are shown in Figure 4. A direct relationship between antimicrobial susceptibility and ability to form biofilm was observed. However, no statistical relationship was found between biofilm formation ability and the antimicrobial resistance classification (*p* = 0.053).

### Identification of Antimicrobial Resistance Genes

In total, 73 (19.4%) isolates were found to be ESBL-producing strains. The most common β-lactamase-encoding gene was *bla*_*CTX–M–*15_ (*n* = 43, 58.9%), followed by *bla*_*TEM–*1_ (*n* = 33, 45.2%), *bla*_*CTX–M–*14_ (*n* = 8, 12.3%), *bla*_*SHV–*1_ (*n* = 7, 9.6%), *bla*_*CTX–M–*27_ (*n* = 5, 6.8%) and *bla*_*CTX–M–*28_ (*n* = 3, 4.1%). None carried the *bla*_*CTX–M–*2_, *bla*_*CTX–M–*8_ or *bla*_*CTX–M–*25_ genes, but 27 strains (37%) harbored more than one β-lactamase-encoding gene.

Regarding biofilm formation, 17 of the ESBLs-producing strains (23.3%) were non-biofilm-formers, 15 (20.5%) were weak biofilm-formers, 25 (34.2%) were moderate biofilm-formers, and 16 (21.9%) were strong biofilm-formers. However, no relationship was found between ESBL production and biofilm formation.

Of the 190 strains resistant to trimethoprim-sulfamethoxazole, 78 (41.1%), 131 (68.9%), and 47 strains (24.7%) harbored *sul1, sul2*, or both genes, respectively.

Among the 188 quinolone-resistant strains, 6 (3.2%) *qnrB* and 40 (21.3%) *aac (6’)-Ib-cr* genes were detected. We observed a higher prevalence of the *aac (6’)-Ib-cr* gene among the non-biofilm-forming strains, being the correlation statistically significant (*p* = 0.008). We detected two colistin-resistant strains, one of which carried the *mcr-1* gene.

### Virulence Determinants Detection

**FIGURE 5:**
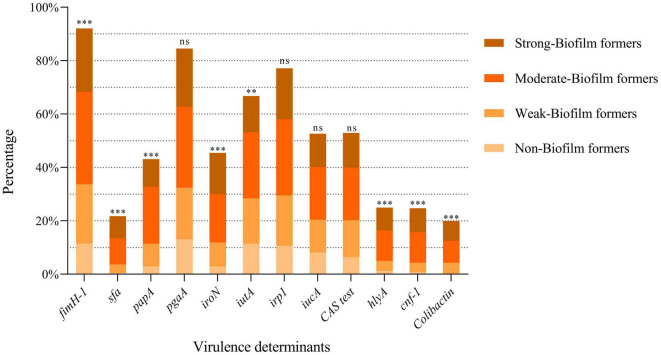
Prevalence of the different VFGs tested according to the biofilm formation ability. Each bar shows the percentage of positivity of each gene within the total strains studied. Within each bar, the percentage according to the ability to form biofilms is shown. ^***^p < 0.001; ^**^p = 0.01; ns: not significant (*p* > 0.05).

The results about the prevalence of the different VFGs tested are shown in Figure 5. It was observed that the prevalence of siderophore-related genes was variable: *irp1* (77.1%), *iutA* (66.8%), *iucA* (52.7%), and *iroN* (45.5%). A significant correlation was found between biofilm formation ability and the presence of *iroN* (*p* < 0.001) or *iutA* (*p* = 0.010) genes. However, *iutA* gene was more prevalent among non-biofilm-forming strains.

The adhesin-encoding genes *fimH* and *pgaA* were the most frequent genes among the strains, with a prevalence of 92% and 84.6%, respectively. The prevalence of the other adhesin-encoding genes, such as *papA* and *sfa*, was 43.1% and 21.8%, respectively. A statistically significant correlation was found between biofilm formation and the presence of the *papA, fimH*, and *sfa* genes (*p* < 0.001).

Among the genes encoding toxins, *hlyA* and *cnf-1* showed a prevalence of 25% and 24.7%, respectively. The *clbA, clbB*, and *clbN* genes were present in 19.9% of the strains. *clbQ* gene was observed in 20.5% of the isolates. It is of note that a statistically significant correlation was confirmed between the presence of toxin-encoding genes and the ability to form biofilm: *hlyA* (*p* = 0.0002), *cnf* (*p* < 0.001), and colibactin-encoding genes (*clbA, clbB, clbN* and *clbQ*) (*p* < 0.001).

### Siderophore Assay (Chrome Azurol S Test)

The 376 isolates were screened on CAS agar plates, a useful method for identifying siderophores in *E. coli* isolates and other Gram-negative bacteria. 199 (52.9%) siderophore-producing strains were found, as shown in Figure 5, but statistical analysis showed no relationship between CAS test positivity and biofilm formation (*p* = 0.132).

On the other hand, a statistically significant association between the positive CAS method and the presence of different siderophore-encoding genes *irp1*, *iucA*, *iutA* (*p* < 0.001) was found. The presence of the fimbriae H coding gene (*fimH*) was also correlated with the CAS test (*p* = 0.025). Contrarily, an association between the CAS test and the absence of the *sfa* gene (*p* = 0.024) was observed.

It is to note that strains resistant to antibiotics such as gentamicin (*p* = 0.032), ampicillin (*p* = 0.001), cefotaxime (*p* = 0.001), ceftazidime (*p* = 0.002), cefepime (*p* = 0.001) or aztreonam (*p* < 0.001) showed high siderophore production (positive CAS test). Likewise, ESBL-producing strains were statistically associated with a positive CAS test (*p* = 0.001). Finally, strains belonged to phylogroup B2 produced more siderophores than strains belonging to the other phylogroups (*p* < 0.001).

### Phylogroup Assignment Method

In our study, the phylogenetic groups considered more virulent B2 and D, accounted for 72.9% of the *E. coli* isolates [B2: *n* = 235 (62.5%); D: *n* = 39 (10.4%)]. The less virulent groups were found in varying percentages [A: *n* = 18 (4.8%); B1: *n* = 31 (8.2%); C: *n* = 18 (4.8%); E: *n* = 4 (1.1%); F: *n* = 26 (6.9%); unknown: *n* = 5 (1.3%)].

**FIGURE 6:**
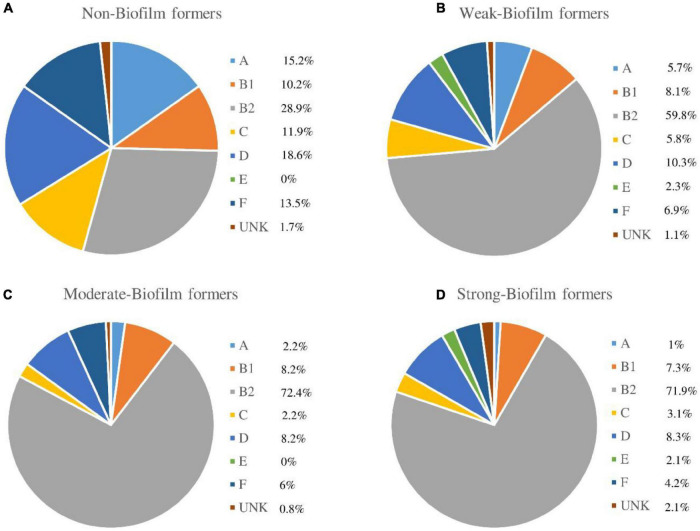
Phylogroup assignment according to the biofilm formation. UNK: Unknown. **(A)** Non-Biofilm former strains. **(B)** Weak-Biofilm former strains. **(C)** Moderate-Biofilm former strains. **(D)** Strong-Biofilm former strains.

According to biofilm classification, a great variety of phylogroups was observed in the non-biofilm-forming strains group. In contrast, a direct relationship between the ability to form biofilm and phylogroup B2 was observed, being this relationship statistically significant (*p* < 0.001) (Figure 6).

### Characterization of Colibactin-Encoding *Escherichia coli* Strains

The presence of *clbA, clbB, clbN*, and *clbQ* genes confirmed the full presence of the *pks*-island in 75 strains. Among these, 74 presented the ability to form biofilm. We compared whether the presence of colibactin-encoding genes was related to biofilm formation ability and we found a statistically significant relationship between these two variables (*p* < 0.001).

As shown in Table 3, colibactin-producing strains showed a high prevalence of most of the VFGs tested (*iroN, irp1, papA, fimH, cnf, hlyA, pgaA*, and *sfa*) (*p* < 0.05), but had a low prevalence of the *iucA* and *iutA* genes. The relationship between susceptibility to most of the antimicrobials tested (amoxicillin/clavulanic acid, ampicillin, aztreonam, cefepime, cefotaxime, ceftazidime, chloramphenicol, ciprofloxacin, gentamicin, and trimethoprim-sulfamethoxazole) and the presence of colibactin genes was statistically significant (*p* < 0.05). Only two of the colibactin-encoding strains were ESBL-producers, showing an inverse correlation between colibactin genes and ESBL production (*p* < 0.001). Most colibactin-positive strains were isolated from urine (*n* = 45; 60%), followed by strains from blood (*n* = 22; 29.3%), and the respiratory tract (*n* = 8; 10.7%), but no correlation was found between the strain source and the presence of colibactin-encoding genes.

**TABLE 3:** Characterization of colibactin-encoding *Escherichia coli* strains.

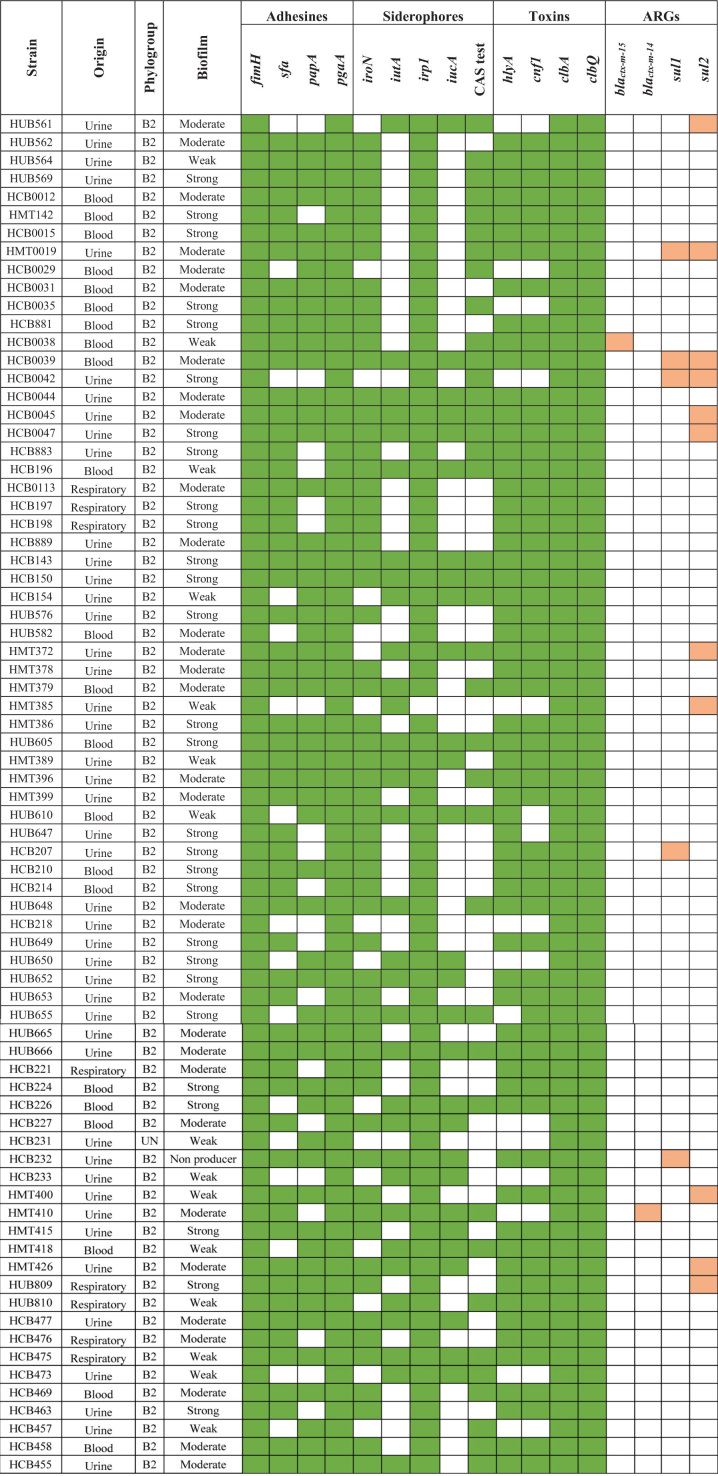

*Colored squares denote the presence of the gene or positivity of the test.*

*ARGs: antimicrobial resistance genes. UN: Unknown.*

**FIGURE 7:**
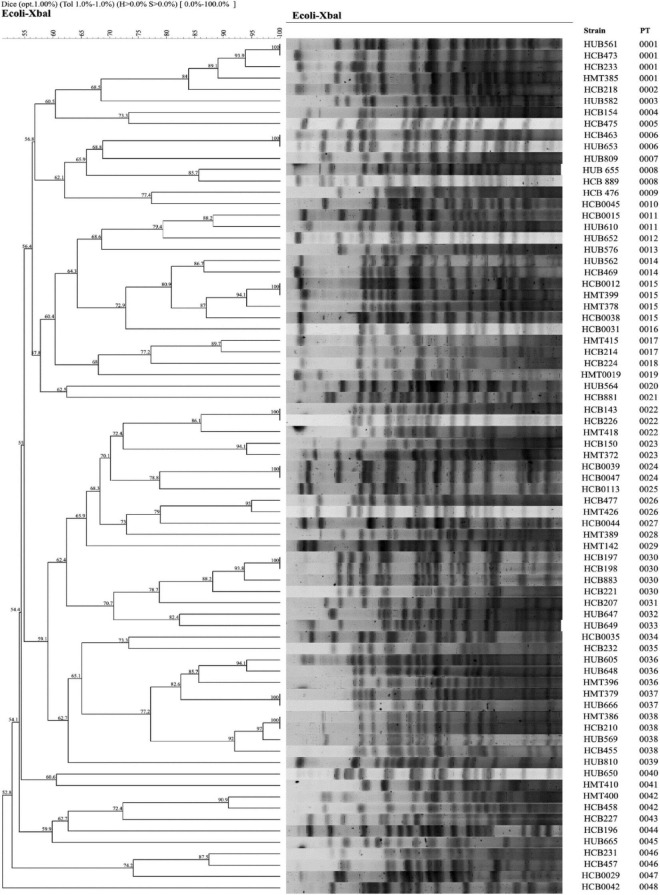
Clonal relationship analysis of the colibactin strains assessed by PFGE using *Xba*I. PT: Pulsotype.

The majority of the colibactin-positive strains belonged to phylogroup B2, and the analysis of the clonal relatedness by PFGE of the colibactin-positive *E. coli* strains showed 48 different clusters with a Dice similarity index ≥ 85%. This indicates that the *E. coli* isolates harboring the colibactin toxin exhibit a high clonal diversity (Figure 7).

## Discussion

Extraintestinal pathogenic *E. coli* strains are a group of bacteria that can cause urinary tract, bloodstream and other non-intestinal infections, both in healthcare settings and in the community (Manges et al., 2019). Several studies have investigated the association between antimicrobial resistance and/or the presence of some VFGs and the ability to form biofilms (May and Okabe, 2011; Friedlander et al., 2015; Cepas et al., 2019). However, the virulence genes described in recent years need to be studied in more detail, as they may be related to biofilm formation. In this study, we characterized 376 strains to investigate the possible relationship between antimicrobial resistance and/or the presence of selected virulence determinants and the ability to form biofilms. Our results showed that almost all the isolates studied (84.3%) were able to form biofilms, and this high rate might be related to some of the genes and features observed among the strains studied.

In terms of antimicrobial resistance, we found high rates of resistance to ciprofloxacin (48.7%), trimethoprim-sulfamethoxazole (47.9%), and ampicillin (38%), and fully susceptible strains to carbapenems (imipenem and meropenem). Comparing our results with the SMART study (Cantón et al., 2021), which investigated Gram-negative bacilli isolated from intra-abdominal, urinary, bloodstream and lower respiratory tract infection from 2016 to 2018 in 11 participating Spanish Hospitals, we found similar resistance rates for cephalosporines, carbapenems and colistin but their antimicrobial resistance rates to ciprofloxacin were lower (32.3%). Besides, they found a lower rate of ESBL-producing strains (8.6%). According to the data from the 2017 European Antimicrobial Resistance Surveillance Network report (European Centre for Disease Prevention and Control [ECDC], 2017), which is based on antimicrobial resistance data from invasive isolates, we found similar percentages of fully susceptible strains but a higher rate of MDR in the collection under study. They also reported the highest average resistance percentage for aminopenicillins (58.7%), followed by fluoroquinolones (25.7%). These differences could be probably due to most of the strains in the present study were isolated from urine, and some of the most common antibiotics prescribed in UTIs are ciprofloxacin and trimethoprim-sulfamethoxazole. Previous studies conducted by our group showed no statistically significant relationship between biofilm formation ability and ciprofloxacin resistance among *E. coli* strains, but this association was found in *Pseudomonas aeruginosa* (Cepas et al., 2019). However, after analyzing whether this association existed in our study, a statistically significant association was found between non-biofilm-forming strains and resistance to ciprofloxacin. These variations among the data could be due to the greater number of strains analyzed in the present research. Several studies reported that quinolone-resistant strains were less virulent than quinolone-susceptible strains, observing that fimbriae genes, associated with biofilm formation, were less prevalent among nalidixic acid-resistant *E. coli* isolates (Vila et al., 2002; Horcajada et al., 2005). Moreover, the study by Fàbrega et al. (2014) showed that the acquisition of quinolone resistance was related to a decrease in biofilm formation in *Salmonella enterica* strains (Fàbrega et al., 2014) which may explain our results.

Regarding ESBL, these were highly prevalent in our study (19.4%) compared to the study conducted by Flament-Simon et al. (2020) in which only 13 of the strains (6.6%) isolated in Spain and France in 2016 produced ESBL enzymes (Flament-Simon et al., 2020). In agreement with our results, several epidemiological studies confirm that *bla*_*CTX–M–*15_ is one the most common ESBL among clinical isolates from Spain. However, *bla*_*CTX–M–*14_ and *bla*_*CTX–M–*27_ have also been found in this country (Dahbi et al., 2014; Merino et al., 2016, 2018). Interestingly, three strains carrying *bla*_*CTX–M–*28_ were found in our study, which is less prevalent than other *bla*_*CTX–M–*1_ enzymes (Livermore et al., 2007). When we analyzed the relation between biofilm formation and ESBL production, we did not find any correlation between these two variables. However, Shrestha et al. (2019) found a positive correlation between these two features (Shrestha et al., 2019). The differences between the two studies could be due to the higher percentage of beta-lactamases (50.9% vs. 19.4%) they found. These results showing a worrying situation, since having a high percentage of ESBL among biofilm-forming strains makes very difficult their eradication.

As for virulence, we found 14 (3.7%) strains carrying all the VFGs tested. However, most were associated with low antimicrobial resistance rates. We observed a statistically significant correlation between the presence of different siderophores, adhesins or toxins and the ability to form biofilms. Siderophores are small molecules with high affinity for iron (Holden and Bachman, 2015). They are considered an important virulence factor in most Gram-negative bacteria. In *S. maltophilia*, iron plays a significant role in biofilm formation and production of EPS (Kalidasan et al., 2018). In *P. aeruginosa*, siderophore-deficient clones showed reduced biofilm formation ability (Banin et al., 2005). In *E. coli*, studies by May and Okabe (2011) have shown that biofilm formation is favored in media with low iron concentrations (May and Okabe, 2011). In support of previous studies, we found a significant correlation between the presence of siderophore-encoding genes, such as *iroN*, and the ability to form biofilms. In addition, resistant strains produced more siderophores than their susceptible counterparts. Recently, it has been reported that the introduction of the mobile genetic element ICEKp from *Klebsiella pneumoniae* or a plasmid encoding YbtPQ (a siderophore importer encoded in the yersiniabactin cluster) reduced the susceptibility of *E. coli* to a wide range of antimicrobials (Farzand et al., 2021). Regarding adhesins, type 1 fimbriae are the best known adhesive organelles among Enterobacteriaceae (Abdelhalim et al., 2020) playing an important role in the initial steps of biofilm formation. As expected, we found a significant association between the presence of the *fimH* gene and biofilm formation. The adhesins *sfa* and *papA* also showed a significant association with biofilm formation. Although poly-β-1,6-N-acetyl-D-glucosamine polymer is associated with biofilm formation by mediating cell-to-cell adhesion and attachment to surfaces (Sharma et al., 2016), our results showed no statistical correlation between the presence of the *pgaA* gene and the capacity to form biofilms.

An important finding of the present work is the high presence of the toxin colibactin among the strains under study. Colibactin is a virulence determinant and a genotoxic enzyme synthesized by polyketide synthases and encoded by a 54-kb genomic island designated *pks* (Faïs et al., 2018). Colibactin may induce DNA damage in the host and has been correlated with colorectal cancer in humans (Wernke et al., 2020). Previous studies have confirmed the presence of *pks*-positive strains among ExPEC (Auvray et al., 2021). Interestingly, 75 of our isolates (19.9%) were colibactin-encoding strains, a higher percentage than this observed by Suresh et al. (2018). Analysis of the association between colibactin and other VFGs showed a significant correlation of this toxin with the siderophore-encoding genes *iroN* and *irp1;* the adhesin-encoding genes *papA, fimH, pgaA*, and *sfa*; and the toxin genes *cnf-1* and *hlyA*. Iron is an essential element for the survival of *E. coli*. Previous studies confirm that the *pks* island is involved in the synthesis of siderophores such as yersiniabactin, enterobactin, and salmochelins via phosphopantetheinyl transferase ClbA (Martin et al., 2013). In addition, it has been suggested that *E. coli* strains carrying hemolysin and colibactin have advantages in colorectal colonization and subsequent cancer (Yoshikawa et al., 2020). As in our case, it has been previously reported that phylogenetic group B2 is predominant among colibactin-encoding *E. coli* strains (Sarshar et al., 2017). However, the high clonal diversity of PFGE analysis among colibactin-positive *E. coli* isolates studied suggests that colibactin may be distributed in a wide variety of strains and clones. Worryingly, Putze et al. (2009) detected colibactin-related genes associated with an ICE-like element in several enterobacteria, not only in *E. coli*, paving the way for the spread of this gene cluster among species (Putze et al., 2009). Furthermore, we observed low rates of resistance to antimicrobial agents among colibactin-positive isolates, which is consistent with previous studies (Sarshar et al., 2017; Morgan et al., 2019). Although colibactin-encoding genes may have a direct influence on other biosynthetic pathways, our findings support the hypothesis that the presence of colibactin-encoding genes may be related to biofilm formation. In this sense, several studies showed a high capacity for biofilm formation among the *pks*-carrying strains studied (Raisch et al., 2014; Suresh et al., 2018). Likewise, Dejea et al. (2014) conducted a study in two geographically distinct cohorts from the United States and Malaysia finding that 89% of cases of right-sided colorectal tumors presented biofilms in the biopsies samples, concluding that a significant association between the presence of biofilms and colorectal cancer exists (Dejea et al., 2014). In another study of patients with familial adenomatous polyposis, the bacterial biofilms found were composed predominantly by *E. coli* and *Bacteroides fragilis*. Genes encoding colibactin (*clbB*) were found at high levels in strains from these patients compared to strains isolated from healthy individuals (Dejea et al., 2018). Additionally, uropathogenic *E. coli* strains carrying the *pks* island have also been described. Chagneau et al. (2021) detected colibactin in 24.7% of urine samples from patients with community-acquired pyelonephritis, cystitis or asymptomatic bacteriuria (Chagneau et al., 2021). They also reported that colibactin was produced during UTIs and induced DNA damage in urothelial cells (Chagneau et al., 2021). Similarly, the study conducted by Morgan and collaborators in an *in vivo* model of ascending UTI in rats, showed that isolates carrying the *cnf-1*, *clbA* and *clbQ* genes induced severe UTIs within 48–72 h (Morgan et al., 2019). Thus, strains carrying colibactin-encoding genes can produce biofilms and cause severe disease not only in the intestine but also in the urinary tract, and probably in other less studied anatomic sites.

## Conclusion

Ciprofloxacin resistance was associated with lower biofilm production ability among the *E. coli* strains under study. Furthermore, biofilm formation ability was correlated with the presence of some siderophores, adhesins, and toxins. The high percentage of strains harboring the colibactin-encoding genes and the significant correlation between this toxin and biofilm formation, suggest that colibactin could be a promising therapeutic target to prevent biofilm formation. Nevertheless, further studies are needed to confirm our hypothesis and to better understand the impact of colibactin production on human health.

## Data Availability Statement

The original contributions presented in the study are included in the article/supplementary material, further inquiries can be directed to the corresponding author.

## Author Contributions

SS and VB: conceptualization and writing—original draft preparation. VB, YG, CR, and MS: methodology. VB: statistical analysis. VB, CR, and SS: writing—review and editing. All authors have read and agreed to the published version of the manuscript.

## Conflict of Interest

The authors declare that the research was conducted in the absence of any commercial or financial relationships that could be construed as a potential conflict of interest.

## Publisher’s Note

All claims expressed in this article are solely those of the authors and do not necessarily represent those of their affiliated organizations, or those of the publisher, the editors and the reviewers. Any product that may be evaluated in this article, or claim that may be made by its manufacturer, is not guaranteed or endorsed by the publisher.

## References

[B1] Abdelhalim K. A. Uzel A. Unal N. G. (2020). The role of major virulence factors and pathogenicity of adherent-invasive *Escherichia coli* in patients with Crohn’s disease. *Prz. Gastroenterol.* 15 279–288. 10.5114/pg.2020.93235 33777266 PMC7988836

[B2] Auvray F. Perrat A. Arimizu Y. Chagneau C. V. Bossuet-Greif N. Massip C. (2021). Insights into the acquisition of the pks island and production of colibactin in the *Escherichia coli* population. *Microb. Genomics* 7:579. 10.1099/mgen.0.000579 33961542 PMC8209727

[B3] Banin E. Vasil M. L. Greenberg E. P. (2005). Iron and *Pseudomonas aeruginosa* biofilm formation. *Proc. Natl. Acad. Sci. USA* 102 11076–11081. 10.1073/pnas.0504266102 16043697 PMC1182440

[B4] Beloin C. Roux A. Ghigo J.-M. (2008). *Escherichia coli* biofilms. *Curr. Top. Microbiol. Immunol.* 322 249–289. 10.1007/978-3-540-75418-3_12 18453280 PMC2864707

[B5] Beloin C. Valle J. Latour-Lambert P. Faure P. Kzreminski M. Balestrino D. (2004). Global impact of mature biofilm lifestyle on *Escherichia coli* K-12 gene expression. *Mol. Microbiol.* 51 659–674. 10.1046/j.1365-2958.2003.03865.x 14731270

[B6] Blount Z. D. (2015). The unexhausted potential of E. coli. *Elife* 4 1–12. 10.7554/eLife.05826 25807083 PMC4373459

[B7] Cantón R. Loza E. Arcay R. M. Cercenado E. Castillo F. J. Cisterna R. (2021). Antimicrobial activity of ceftolozane-tazobactam against Enterobacterales and *Pseudomonas aeruginosa* recovered during the Study for Monitoring Antimicrobial Resistance Trends (SMART) program in Spain (2016-2018). *Rev. Esp. Quimioter.* 34 228–237. 10.37201/req/019.2021 33645948 PMC8179940

[B8] Cepas V. Ballén V. Gabasa Y. Ramírez M. López Y. Soto S. M. (2020). Transposon insertion in the *purL* gene induces biofilm depletion in *Escherichia coli* ATCC 25922. *Pathogens* 9 1–22. 10.3390/pathogens9090774 32971800 PMC7558270

[B9] Cepas V. López Y. Muñoz E. Rolo D. Ardanuy C. Martí S. (2019). Relationship between biofilm formation and antimicrobial resistance in Gram-negative bacteria. *Microb. Drug Resist.* 25 72–79. 10.1089/mdr.2018.0027 30142035

[B10] Chagneau C. V. Massip C. Bossuet-Greif N. Fremez C. Motta J. P. Shima A. (2021). Uropathogenic *E. coli* induces DNA damage in the bladder. *PLoS Pathog.* 17:1–18. 10.1371/JOURNAL.PPAT.1009310 33630958 PMC7906301

[B11] Clermont O. Christenson J. K. Denamur E. Gordon D. M. (2013). The Clermont *Escherichia coli* phylo-typing method revisited: improvement of specificity and detection of new phylo-groups. *Environ. Microbiol. Rep.* 5 58–65. 10.1111/1758-2229.12019 23757131

[B12] CLSI (2020). *CLSI. Performance Standards for Antimicrobial Susceptibility Testing, 30th Edition. CLSI Supplement M100.* Wayne, PA: Clinical and Laboratory Standard Institute.

[B13] Dahbi G. Mora A. Mamani R. Lopez C. Alonso M. P. Marzoa J. (2014). Molecular epidemiology and virulence of *Escherichia coli* O16: H5-ST131: Comparison with H30 and H30-Rx subclones of O25b: H4-ST131. *Int. J. Med. Microbiol.* 304 1247–1257. 10.1016/j.ijmm.2014.10.002 25455219

[B14] Dejea C. Fathi P. Craig J. M. Boleij A. Taddese R. Geis A. L. (2018). Patients with familial adenomatous polyposis harbor colonic biofilms containing tumorigenic bacteria. *Science* 359 592–597. 10.1126/science.aah3648.Patients 29420293 PMC5881113

[B15] Dejea C. M. Wick E. C. Hechenbleikner E. M. White J. R. Mark Welch J. L. Rossettid B. J. (2014). Microbiota organization is a distinct feature of proximal colorectal cancers. *Proc. Natl. Acad. Sci. USA* 111 18321–18326. 10.1073/pnas.1406199111 25489084 PMC4280621

[B16] Dubois D. Delmas J. Cady A. Robin F. Sivignon A. Oswald E. (2010). Cyclomodulins in urosepsis strains of *Escherichia coli*. *J. Clin. Microbiol.* 48 2122–2129. 10.1128/JCM.02365-09 20375237 PMC2884489

[B17] Durmaz R. Otlu B. Koksal F. Hosoglu S. Ozturk R. Ersoy Y. (2009). The optimization of a rapid pulsed-field gel electrophoresis protocol for the typing of *Acinetobacter baumannii*, *Escherichia coli* and Klebsiella spp. *Jpn. J. Infect. Dis.* 62 372–377. 19762987

[B18] Dziubańska-Kusibab P. J. Berger H. Battistini F. Bouwman B. A. M. Iftekhar A. Katainen R. (2020). Colibactin DNA-damage signature indicates mutational impact in colorectal cancer. *Nat. Med.* 26 1063–1069. 10.1038/s41591-020-0908-2 32483361

[B19] European Centre for Disease Prevention and Control (ECDC) (2017). *Antimicrobial resistance surveillance in Europe 2016. Annual Report of the European Antimicrobial Resistance Surveillance Network (EARS-Net).* Solna Municipality: ECDC.

[B20] Fàbrega A. Soto S. M. Ballesté-Delpierre C. Fernández-Orth D. Jiménez de Anta M. T. Vila J. (2014). Impact of quinolone-resistance acquisition on biofilm production and fitness in *Salmonella enterica*. *J. Antimicrob. Chemother.* 69 1815–1824. 10.1093/jac/dku078 24706735

[B21] Faïs T. Delmas J. Barnich N. Bonnet R. Dalmasso G. (2018). Colibactin: More than a new bacterial toxin. *Toxins* 10 16–18. 10.3390/toxins10040151 29642622 PMC5923317

[B22] Farzand R. Rajakumar K. Barer M. R. Freestone P. P. E. Mukamolova G. V. Oggioni M. R. (2021). A virulence associated siderophore importer reduces antimicrobial susceptibility of *Klebsiella pneumoniae*. *Front. Microbiol.* 12:1–9. 10.3389/fmicb.2021.607512 33584611 PMC7876324

[B23] Flament-Simon S. C. Nicolas-Chanoine M. H. García V. Duprilot M. Mayer N. Alonso M. P. (2020). Clonal structure, virulence factor-encoding genes and antibiotic resistance of *Escherichia coli*, causing urinary tract infections and other extraintestinal infections in humans in spain and france during 2016. *Antibiotics* 9 1–17. 10.3390/antibiotics9040161 32260467 PMC7235800

[B24] Friedlander R. S. Vogel N. Aizenberg J. (2015). Role of flagella in adhesion of *Escherichia coli* to abiotic surfaces. *Langmuir* 31 6137–6144. 10.1021/acs.langmuir.5b00815 25945399

[B25] Guerra B. Junker E. Miko A. Helmuth R. Mendoza M. C. (2004). Characterization and localization of drug resistance determinants in multidrug-resistant, integron-carrying *Salmonella enterica* Serotype Typhimurium strains. *Microb. Drug Resist.* 10 83–91. 15256022 10.1089/1076629041310136

[B26] Guerrieri C. G. Pereira M. F. Galdino A. C. M. Dos Santos A. L. S. Elias W. P. Schuenck R. P. (2019). Typical and atypical enteroaggregative *Escherichia coli* are both virulent in the *Galleria mellonella* model. *Front. Microbiol.* 10:1791. 10.3389/fmicb.2019.01791 31456762 PMC6700222

[B27] Guttenplan S. B. Kearns D. B. (2013). Regulation of flagellar motility during biofilm formation. *FEMS Microbiol. Rev.* 37 849–871. 10.1111/1574-6976.12018 23480406 PMC3718880

[B28] Holden V. I. Bachman M. A. (2015). Diverging roles of bacterial siderophores during infection. *Metallomics* 7 986–995. 10.1039/c4mt00333k 25745886

[B29] Horcajada J. P. Soto S. Gajewski A. Smithson A. Jiménez De Anta M. T. Mensa J. (2005). Quinolone-resistant uropathogenic *Escherichia coli* strains from phylogenetic group B2 have fewer virulence factors than their susceptible counterparts. *J. Clin. Microbiol.* 43 2962–2964. 10.1128/JCM.43.6.2962-2964.2005 15956432 PMC1151912

[B30] Houdouin V. Bonacorsi S. Bidet P. Bingen-Bidois M. Barraud D. Bingen E. (2006). Phylogenetic background and carriage of pathogenicity island-like domains in relation to antibiotic resistance profiles among *Escherichia coli* urosepsis isolates. *J. Antimicrob. Chemother.* 58 748–751. 10.1093/jac/dkl326 16905527

[B31] Johnson J. R. Stell A. L. (2000). Extended virulence genotypes of *Escherichia coli* strains from patients with urosepsis in relation to phylogeny and host compromise. *J. Infect. Dis.* 181 261–272. 10.1086/315217 10608775

[B32] Johnson J. R. Johnston B. Kuskowski M. A. Nougayrede J. P. Oswald E. (2008). Molecular epidemiology and phylogenetic distribution of the *Escherichia coli pks* genomic island. *J. Clin. Microbiol.* 46 3906–3911. 10.1128/JCM.00949-08 18945841 PMC2593299

[B33] Johnson J. R. Russo T. A. Tarr P. I. Carlino U. Bilge S. S. Vary J. C. (2000). Molecular epidemiological and phylogenetic associations of two novel putative virulence genes, *iha* and *iroN*_*E. coli*_, among *Escherichia coli* isolates from patients with urosepsis. *Infect. Immun.* 68 3040–3047. 10.1128/IAI.68.5.3040-3047.2000 10769012 PMC97527

[B34] Kalidasan V. Joseph N. Kumar S. Awang Hamat R. Neela V. K. (2018). Iron and virulence in *Stenotrophomonas maltophilia*: All We Know So Far. *Front. Cell Infect. Microbiol.* 8:401. 10.3389/fcimb.2018.00401 30483485 PMC6240677

[B35] Leflon-Guibout V. Jurand C. Bonacorsi S. Espinasse F. Guelfi M. C. Duportail F. (2004). Emergence and spread, of three clonally related virulent isolates of CTX-M-15-producing *Escherichia coli* with variable resistance to aminoglycosides and tetracycline in a French geriatric hospital. *Antimicrob. Agents Chemother.* 48 3736–3742. 10.1128/AAC.48.10.3736-3742.2004 15388428 PMC521882

[B36] Lescat M. Poirel L. Nordmann P. (2018). Rapid multiplex polymerase chain reaction for detection of *mcr-1* to *mcr-5* genes. *Diagn. Microbiol. Infect. Dis.* 92 267–269. 10.1016/j.diagmicrobio.2018.04.010 30220493

[B37] Livermore D. M. Canton R. Gniadkowski M. Nordmann P. Rossolini G. M. Arlet G. (2007). CTX-M: Changing the face of ESBLs in Europe. *J. Antimicrob. Chemother.* 59 165–174. 10.1093/jac/dkl483 17158117

[B38] Magiorakos A. P. Srinivasan A. Carey R. B. Carmeli Y. Falagas M. E. Giske C. G. (2012). Multidrug-resistant, extensively drug-resistant and pandrug-resistant bacteria: An international expert proposal for interim standard definitions for acquired resistance. *Clin. Microbiol. Infect.* 18 268–281. 10.1111/j.1469-0691.2011.03570.x 21793988

[B39] Manges A. R. Geum H. M. Guo A. Edens T. J. Fibke C. D. Pitout J. D. D. (2019). Global extraintestinal pathogenic *Escherichia coli* (ExPEC) lineages. *Clin. Microbiol. Rev.* 32 1–25. 10.1128/CMR.00135-18 PMC6589867 31189557

[B40] Martin P. Marcq I. Magistro G. Penary M. Garcie C. Payros D. (2013). Interplay between siderophores and colibactin genotoxin biosynthetic pathways in *Escherichia coli*. *PLoS Pathog.* 9:3437. 10.1371/JOURNAL.PPAT.1003437 23853582 PMC3708854

[B41] May T. Okabe S. (2011). Enterobactin is required for biofilm development in reduced-genome *Escherichia coli*. *Environ. Microbiol.* 13 3149–3162. 10.1111/j.1462-2920.2011.02607.x 21980953

[B42] Merino I. Hernández-García M. Turrientes M. C. Pérez-Viso B. López-Fresneña N. Diaz-Agero C. (2018). Emergence of ESBL-producing *Escherichia coli* ST131-C1-M27 clade colonizing patients in Europe. *J. Antimicrob. Chemother.* 73 2973–2980. 10.1093/jac/dky296 30124851

[B43] Merino I. Shaw E. Horcajada J. P. Cercenado E. Mirelis B. Pallarés M. A. (2016). CTX-M-15-H30Rx-ST131 subclone is one of the main causes of healthcare-associated ESBL-producing *Escherichia coli* bacteraemia of urinary origin in Spain. *J. Antimicrob. Chemother.* 71 2125–2130. 10.1093/jac/dkw133 27494832

[B44] Monstein H. J. Ostholm-Balkhed A. Nilsson M. V. Nilsson M. Dornbusch K. Nilsson L. E. (2007). Multiplex PCR amplification assay for the detection of *bla*_*SHV*_, *bla*_*TEM*_ and *bla*_*CTX–M*_ genes in *Enterobacteriaceae*. *Apmis* 115 1400–1408. 10.1111/j.1600-0463.2007.00722.x 18184411

[B45] Morgan R. N. Saleh S. E. Farrag H. A. Aboulwafa M. M. (2019). Prevalence and pathologic effects of colibactin and cytotoxic necrotizing factor-1 (Cnf 1) in *Escherichia coli*: Experimental and bioinformatics analyses. *Gut. Pathog.* 11 1–18. 10.1186/s13099-019-0304-y 31139264 PMC6525971

[B46] Pai H. Choi E. H. Lee H. J. JungYun H. Jacoby G. A. (2001). Identification of CTX-M-14 extended-spectrum β-lactamase in clinical isolates of *Shigella sonnei*. *Escherichia coli*, and *Klebsiella pneumoniae* in Korea. *J. Clin. Microbiol.* 39 3747–3749. 10.1128/JCM.39.10.3747-3749.2001 11574608 PMC88424

[B47] Park C. H. Robicsek A. Jacoby G. A. Sahm D. Hooper D. C. (2006). Prevalence in the United States of *aac(6’)-Ib-cr* encoding a ciprofloxacin-modifying enzyme. *Antimicrob. Agents Chemother.* 50 3953–3955. 10.1128/AAC.00915-06 16954321 PMC1635235

[B48] Putze J. Hennequin C. Nougayrède J. P. Zhang W. Homburg S. Karch H. (2009). Genetic structure and distribution of the colibactin genomic island among members of the family *Enterobacteriaceae*. *Infect. Immun.* 77 4696–4703. 19720753 10.1128/IAI.00522-09 PMC2772509

[B49] Raisch J. Buc E. Bonnet M. Sauvanet P. Vazeille E. de Vallée A. (2014). Colon cancer-associated B2 *Escherichia coli* colonize gut mucosa and promote cell proliferation. *World J. Gastroenterol.* 20 6560–6572. 10.3748/wjg.v20.i21.6560 24914378 PMC4047342

[B50] Robicsek A. Strahilevitz J. Jacoby G. A. Macielag M. Abbanat D. Chi H. P. (2006). Fluoroquinolone-modifying enzyme: a new adaptation of a common aminoglycoside acetyltransferase. *Nat. Med.* 12 83–88. 10.1038/nm1347 16369542

[B51] Sáez-López E. Bosch J. Salvia M. D. Fernández-Orth D. Cepas V. Ferrer-Navarro M. (2017). Outbreak caused by *Escherichia coli* O18:K1:H7 sequence type 95 in a neonatal intensive care unit in Barcelona, Spain. *Pediatr. Infect. Dis. J.* 36 1079–1086. 10.1097/INF.0000000000001652 28650938

[B52] Sarowska J. Futoma-Koloch B. Jama-Kmiecik A. Frej-Madrzak M. Ksiazczyk M. Bugla-Ploskonska G. (2019). Virulence factors, prevalence and potential transmission of extraintestinal pathogenic *Escherichia coli* isolated from different sources: Recent reports. *Gut. Pathog.* 11 1–16. 10.1186/s13099-019-0290-0 30828388 PMC6383261

[B53] Sarshar M. Scribano D. Marazzato M. Ambrosi C. Aprea M. R. Aleandri M. (2017). Genetic diversity, phylogroup distribution and virulence gene profile of pks positive *Escherichia coli* colonizing human intestinal polyps. *Microb. Pathog.* 112 274–278. 10.1016/j.micpath.2017.10.009 28987619

[B54] Schwyn B. Neilands J. B. (1987). Universal chemical assay for the detection and determination of siderophores. *Anal. Biochem.* 160 47–56. 10.1016/0003-2697(87)90612-9 2952030

[B55] Sharma G. Sharma S. Sharma P. Chandola D. Dang S. Gupta S. (2016). *Escherichia coli* biofilm: development and therapeutic strategies. *J. Appl. Microbiol.* 121 309–319. 10.1111/jam.13078 26811181

[B56] Shrestha R. Khanal S. Poudel P. Khadayat K. Ghaju S. Bhandari A. (2019). Extended spectrum β-lactamase producing uropathogenic *Escherichia coli* and the correlation of biofilm with antibiotics resistance in Nepal. *Ann. Clin. Microbiol. Antimicrob.* 18 1–6. 10.1186/s12941-019-0340-y 31847837 PMC6918583

[B57] Smith D. R. Price J. E. Burby P. E. Blanco L. P. Chamberlain J. Chapman M. R. (2017). The production of curli amyloid fibers is deeply integrated into the biology of *Escherichia coli*. *Biomolecules* 7:75. 10.3390/biom7040075 29088115 PMC5745457

[B58] Stepanović S. Vuković D. Hola V. Di Bonaventura G. Djukić S. Ćirković I. (2007). Quantification of biofilm in microtiter plates: Overview of testing conditions and practical recommendations for assessment of biofilm production by staphylococci. *Apmis* 115 891–899. 10.1111/j.1600-0463.2007.apm_630.x 17696944

[B59] Suresh A. Ranjan A. Jadhav S. Hussain A. Shaik S. Alam M. (2018). Molecular genetic and functional analysis of *pks*-harboring, extra-intestinal pathogenic *Escherichia coli* from India. *Front. Microbiol.* 9:1–8. 10.3389/fmicb.2018.02631 30498477 PMC6249908

[B60] Takahashi A. Kanamaru S. Kurazono H. Kunishima Y. Tsukamoto T. Ogawa O. (2006). *Escherichia coli* isolates associated with uncomplicated and complicated cystitis and asymptomatic bacteriuria possess similar phylogenies, virulence genes, and O-serogroup profiles. *J. Clin. Microbiol.* 44 4589–4592. 10.1128/JCM.02070-06 17065267 PMC1698404

[B61] Van Gerven N. Van der Verren S. E. Reiter D. M. Remaut H. (2018). The role of functional amyloids in bacterial virulence. *J. Mol. Biol.* 430 3657–3684. 10.1016/j.jmb.2018.07.010 30009771 PMC6173799

[B62] Vila J. Simon K. Ruiz J. Horcajada J. P. Velasco M. Barranco M. (2002). Are quinolone-resistant uropathogenic *Escherichia coli* less virulent? *J. Infect. Dis.* 186 1039–1042. 12232848 10.1086/342955

[B63] Wernke K. M. Xue M. Tirla A. Kim C. S. Crawford J. M. Herzon S. B. (2020). Structure and bioactivity of colibactin. *Bioorganic Med. Chem. Lett.* 30:127280. 10.1016/j.bmcl.2020.127280 32527463 PMC7309967

[B64] Wiegand I. Geiss H. K. Mack D. Stu E. Seifert H. Icrobiol J. C. L. I. N. M. (2007). Detection of Extended-Spectrum Beta-Lactamases among *Enterobacteriaceae* by use of semiautomated microbiology systems and manual detection procedures. *J. Clin. Microbiol.* 45 1167–1174. 10.1128/JCM.01988-06 17287329 PMC1865808

[B65] Woodford N. Fagan E. J. Ellington M. J. (2006). Multiplex PCR for rapid detection of genes encoding CTX-M extended-spectrum β-lactamases [4]. *J. Antimicrob. Chemother.* 57 154–155. 10.1093/jac/dki412 16284100

[B66] Xu J. G. Cheng B. Wen X. Cui S. Ye C. (2000). High-pathogenicity island of *Yersinia* spp. in *Escherichia coli* strains isolated from diarrhea patients in China. *J. Clin. Microbiol.* 38 4672–4675. 10.1128/jcm.38.12.4672-4675.2000 11101622 PMC87663

[B67] Yoshikawa Y. Tsunematsu Y. Matsuzaki N. Hirayama Y. Higashiguchi F. Sato M. (2020). Characterization of colibactin-producing *Escherichia coli* isolated from Japanese patients with colorectal cancer. *Japanese J. Infect. Dis.* 73 437–442. 10.7883/yoken.JJID.2020.066 32475872

